# The Safety and Immunogenicity of GTU^®^MultiHIV DNA Vaccine Delivered by Transcutaneous and Intramuscular Injection With or Without Electroporation in HIV-1 Positive Subjects on Suppressive ART

**DOI:** 10.3389/fimmu.2019.02911

**Published:** 2019-12-13

**Authors:** G. Haidari, Suzanne Day, M. Wood, H. Ridgers, Alethea V. Cope, Sue Fleck, Celine Yan, Kalevi Reijonen, Drew Hannaman, Aggeliki Spentzou, Peter Hayes, A. Vogt, Behazine Combadiere, Adrian Cook, Sheena McCormack, Robin J. Shattock

**Affiliations:** ^1^Group of Mucosal Infection and Immunity, Department of Medicine, Imperial College London, London, United Kingdom; ^2^London School of Hygiene and Tropical Medicine, London, United Kingdom; ^3^FIT Biotech Ltd., Tampere, Finland; ^4^Ichor Medical Systems Inc, San Diego, CA, United States; ^5^Human Immunology Laboratory, International AIDS Vaccine Initiative, Imperial College London, London, United Kingdom; ^6^Department of Dermatology and Allergy, Clinical Research Center for Hair and Skin Science, Charité - Universitätsmedizin Berlin, Berlin, Germany; ^7^Sorbonne Université, Centre d'Immunologie et des Maladies Infectieuses (CIMI-Paris), INSERM U1135, Paris, France; ^8^Medical Research Council Clinical Trials Unit at UCL, University College London, London, United Kingdom

**Keywords:** HIV-1, therapeutic vaccine, electroporation, transcutaneous, plasmid DNA

## Abstract

Previous studies have shown targeting different tissues via the transcutaneous (TC) and intramuscular injection (IM) with or without electroporation (EP) has the potential to trigger immune responses to DNA vaccination. The CUTHIVTHER 001 Phase I/II randomized controlled clinical trial was designed to determine whether the mode of DNA vaccination delivery (TC+IM or EP+IM) could influence the quality and function of induced cellular immune responses compared to placebo, in an HIV positive clade B cohort on antiretroviral therapy (ART). The GTU^®^MultiHIV B DNA vaccine DNA vaccine encoded a MultiHIV B clade fusion protein to target the cellular response. Overall the vaccine and regimens were safe and well-tolerated. There were robust pre-vaccination IFN-γ responses with no measurable change following vaccination compared to placebo. However, modest intracellular cytokine staining (ICS) responses were seen in the TC+IM group. A high proportion of individuals demonstrated potent viral inhibition at baseline that was not improved by vaccination. These results show that HIV positive subjects with nadir CD4+ counts ≥250 on suppressive ART display potent levels of cellular immunity and viral inhibition, and that DNA vaccination alone is insufficient to improve such responses. These data suggest that more potent prime-boost vaccination strategies are likely needed to improve pre-existing responses in similar HIV-1 cohorts (This study has been registered at http://ClinicalTrials.gov under registration no. NCT02457689).

## Introduction

The development of safe and effective antiretroviral therapy (ART) has led to almost near normal life expectancy in people living with HIV, if diagnosed early and established on treatment (1). However, ART whilst able to control infection cannot eradicate the viral reservoir, as evidenced by viral rebound after treatment interruption (2, 3). Additional approaches are required to provide long-term remission from viral rebound during treatment interruption.

The rationale for the development of a therapeutic HIV-1 vaccine is based on several immunological factors. These include the inability of natural infection with HIV to elicit sufficient immunity to control replication, HIV infection itself causing major abnormalities in the cellular immune response and the failure of ART to eradicate established infection (4). A successful therapeutic vaccine needs to induce a broad immune response, modify the host immune response, and help eradicate the reservoir (5). Several combination strategies are currently in progress including the use of a vaccine plus latency reversal agent known as the “shock and kill” approach (6, 7), gene therapy with modification of CCR5 expression on target cells (8), and the use of broadly neutralizing antibodies to induce ART-free viral suppression (9).

The CUTHIVTHER 001 trial was a placebo controlled randomized trial designed to explore the safety and immunogenicity of 2 different delivery modes of the GTU^®^MultiHIV B DNA vaccine in HIV clade B positive volunteers on established ART. Data from a previous study, performed in South African clade C infected volunteers (not on ART) using the same DNA vaccine showed that both intramuscular (IM) and intradermal (ID) delivery was safe and well-tolerated with no vaccine-related serious adverse events (SAEs) (10). A modest but significantly lower log pHIV-RNA was seen in subjects receiving vaccine compared to placebo (*p* = 0.012) with a trend toward higher CD4+ T cell counts (*p* = 0.066). These changes were more pronounced after IM administration and in some HLA haplotypes (B^*^5703) and maintained for 17 months after the final immunization. Given this clade B vaccine was not matched to the predominant clade C epidemic in Sub-Saharan Africa, it is reasonable to expect it to be more effective when tested in a clade matched cohort and that this could be further enhanced using electroporation (EP).

More recent data from our previous clinical trial in HIV negative volunteers (CUTHIVAC 001) demonstrated that IM+EP delivery of the same vaccine promoted strong IFN-γ responses with potent viral inhibition compared to standard delivery by IM+ID (11). Only one other trial to date has used IM+EP with DNA in the context of therapeutic vaccination and showed enhanced CD4+ but not CD8+ T cell responses (12). By contrast TC+IM (without EP) shifted responses toward a more Th-17 dominated phenotype associated with mucosal protection. In this respect, TC vaccination, mediated by targeted DNA delivery via hair follicles following cyanoacrylate skin surface stripping, represents a novel route of immunization. To the best of our knowledge, this is the first trial using TC vaccination with DNA in a HIV positive cohort.

## Materials and Methods

### Trial Design

The CUTHIVTHER 001 was a Phase I/II randomized controlled clinical trial to investigate the safety and immunogenicity of the GTU^®^MultiHIV B clade DNA vaccine administered 3 times in people living with clade B HIV infection who were stable on antiretroviral therapy (ART). This was defined as a viral load of <50 copies/ml on 2 separate occasions in the last 6 months prior to enrolment, nadir CD4 ≥ 250, and screening CD4 ≥200 (Supplementary Table 2.1). The vaccine was administered either IM and enhanced with EP, or IM with TC delivery at 0, 4, and 12 weeks (Supplementary Figure 2.1). A placebo group was included in anticipation of high background responses to peptide pools in a HIV-infected cohort of participants. Participants were randomized to receive either IM+EP or IM+TC vaccination with a further randomization to receive placebo (normal saline) or vaccine. The primary safety endpoint was a grade 3 (severe) local or systemic reaction or an adverse event that led to a clinical decision to discontinue vaccination. The primary immunogenicity endpoint was a doubling in IFN-γ ELISpot response to any of the vaccine peptides between week 0 and week 14.

### Ethics

The CUTHIVTHER 001 trial was conducted in compliance with UK Clinical Trial Regulations and within the principles of Good Clinical Practice (GCP). The study was approved by the National Research Ethics Service, York North East Research Ethics Committee (4/NE/1246, Eudract 2013-004023-37) and by the Medicines and Healthcare products Regulatory Authority (MHRA). All participants provided written informed consent after thorough counseling by the clinical trial team.

### Intervention

The investigational GTU^®^MultiHIV B clade DNA vaccine is a synthetic fusion protein comprising of full-length polypeptides of *Rev, Nef, Tat, p17/p24*, and *CTL* (containing epitopes of protease, reverse transcriptase, and gp160) regions of the primary HAN-2 HIV B clade virus. This vector developed by FIT Biotech is a non-replicating expression vector with enhanced features provided by the bovine papilloma virus transcriptional activators and segregation/partitioning factor E2 protein along with its multimeric specific sites (13).

### Transcutaneous Vaccination

0.2 ml (0.4 mg) of DNA vaccine was administered by the TC route on the external aspect of the upper left arm below the deltoid muscle as previously described (11). In brief, a total amount of 190 mg (equivalent to 9 drops) of cyanoacrylate glue was applied over the investigational site (16 cm^2^), followed by the application of adhesive tape. After hardening of the glue for 20 min, the tape and glue were removed from the skin surface. In order to prevent uncontrolled spreading and loss of vaccine, a rectangular silicone frame was then taped onto the investigational site. The vaccine was then applied drop-wise and spread using a finger cot over the investigational site. This application procedure was followed by an incubation time of 20 min after which a hydrocolloid bandage was placed over the arm for 24 h. TC application was followed by IM injection of 1 ml (2 mg) of DNA vaccine into the vastus lasteralis muscle as described below (Supplementary Data 1.1).

### Intramuscular Injection With Electroporation

1 ml (2 mg) of DNA vaccine was loaded into a disposable electroporation cartridge and delivered IM followed by electroporation using the same hand-held delivery system into the vastus lasteralis muscle of the left leg (TriGrid Delivery systems, San Diego, CA). This method has been described previously (14) (Supplementary Data 1.2).

### IFN-γ ELISpot Assay

IFN-γ ELISpot assays were performed using frozen isolated peripheral blood mononuclear cells stimulated with peptide pools (see Supplementary Table 2.9) matched to the GTU^®^MultiHIV B clade DNA vaccine using a technique previously described (15). An additional integrase peptide pool was added as a control to the assay as the presence of a peptide pool not included in the vaccine may give information on any non-specific changes occurring within T cells related to vaccination (Supplementary Data 1.3). All samples were run in triplicate.

### Human Leucocyte Antigen (HLA) Typing

Blood was taken at week 0 and high and low resolution HLA class I typing was performed by sequence specific PCR according to standard protocols at the Department of Histocompatibility and Immunogenetics, Hammersmith Hospital. The final HLA I typing results is presented as “interpreted” HLA type combining high and low resolution PCR.

### Viral Inhibition Assay

This assay was based on a modified version of a previously described assay (16).

In the endogenous Viral inhibition assay, CD4+ cells were not infected with exogenous virus, but endogenous virus was allowed to replicate during the cell expansion phase of the assay. The Viral inhibition assay was conducted at (week 0) and post vaccination (week 14) to determine whether endogenous virus was inhibited.

For the exogenous Viral inhibition assay, endogenous virus was first blocked using the CCR5 inhibitor Maraviroc by adding 10 μM to the culture on days 3 and 6. The Viral inhibition assay was then conducted at pre (week 0) and post (week 14) vaccination time points as per previously described. Three exogenous viruses were used in this part of the assay; IIIB (clade B), U455 (clade A), and CBL4 (clade D) to determine clade specific and cross clade activity (Supplementary Data 1.4). All samples were run in quadruplicate.

### Intracellular Cytokine Analysis

Intracellular cytokine staining (ICS) was performed on PBMC isolated on pre-vaccination (week 0), 2 weeks post-third vaccination (week 14), and 8 weeks post-third vaccination (week 20) with a panel for CD8+ and CD4+ antigen-specific responses. Briefly, overlapping peptide pools matching the vaccine (Gag, Nef, Rev, Tat and CTL) were incubated with PBMC for 6 h before staining CD4+/CD8+ T cells for CD107a BUV395, IFN-γ AF488, TNF-α PE-Cy7, IL-2 BV510, CD154 BV421, Granzyme B AF647, and Perforin PE. PMA/Ionomycin and integrase peptide pool were used as positive controls. Samples were fixed prior to flow cytometry analysis on a Becton Dickinson Fortessa LSRII. Single samples were run for each condition, all data was analyzed using FlowJo and boolean gated to show polyfunctionality (Supplementary Data 1.5).

### Statistical Analysis

All clinical and routine laboratory data is included in the safety and immunogenicity analysis. Safety data are analyzed as intention to treat. For the immunogenicity analysis the primary endpoint was defined as 2 weeks after the third and final vaccine (week 14). The difference in magnitude of the IFN-γ response between the groups at the specified time points including the primary endpoint for each peptide was compared using an unpaired non-parametric *t* test (Mann-Whitney test). Results were considered statistically significant if the 2 tailed *P* value was <0.05. For the analysis of the difference within each individual group week 0 and week 14, a paired *t* test (Wilcoxon matched-pairs signed rank test) was used where statistical significance was set at *p* = <0.05.

## Results

### GTU®MultiHIV B Clade DNA Vaccine Is Safe and Well-Tolerated in HIV Positive Adults When Delivered by TC+IM and EP+IM Routes

In total 36 participants were screened, and 30 met the eligibility criteria (Supplementary Table 2.1). All but 1 participant completed the vaccination schedule. This participant was in the EP+IM (active arm) and was diagnosed with acute hepatitis C following the second vaccine, and subsequently did not receive the final vaccine but remained in follow up and is included in all analyses (Supplementary Figure 2.2).

All participants enrolled were male with a median age of 34 years. 90% were White and 83% in full time employment. The median number of years since diagnosis of HIV was 3 years, and participants had nadir CD4+ counts ≥250 (median 346; range 260–968 cells/mm^3^). To be enrolled in the study all participants had to be on ART for at least 6 months, and the mean number of years on ART was 2.18 years with a combination of regimens, the most common of which was triple therapy with an NRTI plus NNRTI (Supplementary Table 2.3).

Overall the GTU^®^MultiHIV B clade DNA vaccine was well-tolerated compared to placebo. There were no serious adverse events and only one primary endpoint (the participant who acquired hepatitis C). The majority (73) of 82 unsolicited adverse events (AEs) were mild in nature with the remaining 9 graded moderate (Supplementary Table 2.4). Of note 7 participants in the TC+IM group developed hyper or hypopigmentation (6 active;1 placebo), all of which had resolved by the end of the study.

The EP tolerability data collected from participants after each vaccination suggested no difference in pain scores in those receiving active drug or placebo. All participants deemed the EP procedure to be acceptable both in the context of HIV prevention and treatment (Supplementary Table 2.5).

### IFN-γ ELISpot: Participants Demonstrated Robust Pre-vaccination IFN-γ Responses That Were Not Improved at the Primary Endpoint Either Between or Within Groups

**Figure 1:**
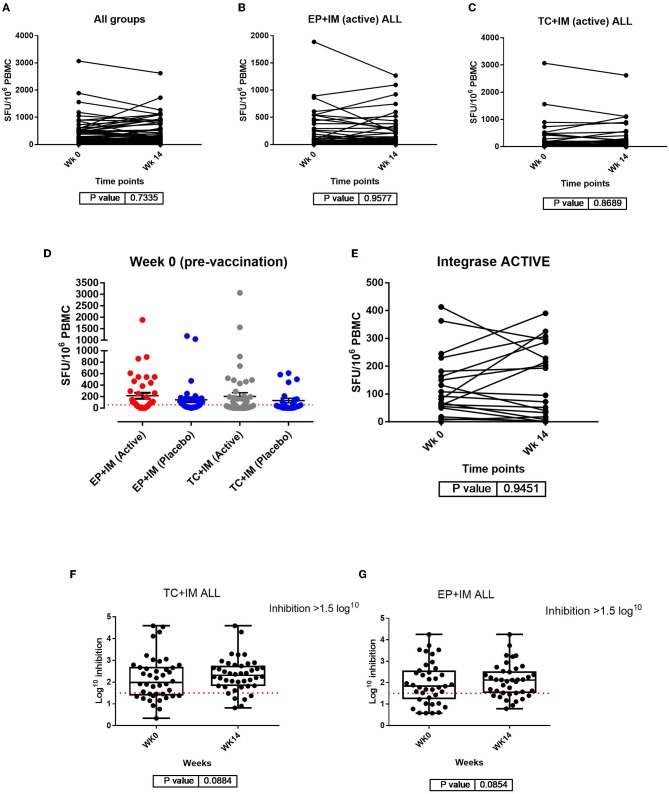
Analysis of IFN-γ responses by ELISpot: **(A)** Comparison of IFN-ɤ ELISpot responses pre vaccination and at the primary endpoint for all participants (both groups); **(B)** Comparison of IFN-ɤ response from week 0 to week 14 with all peptide pools together for the EP+IM group (active); **(C)** Comparison of IFN-ɤ response from week 0 to week 14 with all peptide pools together for the TC+IM group (active) to all peptide pools. Statistical significance is set at *p* = <0.05 (Wilcoxon matched-pairs signed *t* test). Each dot joined together by a line is an individual's IFN-ɤ response at week 0 and 14. **(D)** Scatter dot plot showing baseline IFN-ɤ response at week 0 (pre vaccination) by group. All units are in SFU/M PBMC with background (from negative/mock wells) subtracted. The red dotted line represents the cut off of >55 SFU/M but participants also had to meet the second criteria to be a positive responder (>4 x baseline response if baseline response is more than 0). Viral inhibition assay: **(E)** IFN-ɤ ELISpot responses to Integrase peptide for both groups receiving active drug at week 0 and week 14. **(F)** Comparison of viral inhibition (log^10^) between week 0 and week 14 in the TC+IM group (including placebo). **(G)** Comparison of viral inhibition between week 0 and week 14 in the EP+IM group (including placebo). The red line represents the cut off for positive inhibition of >1.5 log^10^. Statistical significance set at *p* = < 0.05.

A comparison of the mean IFN-γ response by ELISpot at week 0 and the primary endpoint at week 14 shows little variability in SFU/M PBMC with no significant difference comparing data from all groups at week 0 and week 14 (*p* = 0.7335). Furthermore, a comparison of the IFN-γ response within each group at week 0 and week 14 showed no significant difference for any peptide at the primary endpoint in either the EP+IM (active) group or TC+IM (active) group (Figures 1A–C).

Variability of pre-vaccination responses (week 0) was noted in this trial and has been documented in other HIV-1 therapeutic vaccine trials (17). In particular, 24/30 (80%) participants had responses to *Nef*, and 26/30 (87%) to *Gag* and 12/30 (40%) to *CTL* peptide pools respectively, with some IFN-γ responses above the “positive” cut-off of >55 SFU/M PBMC (Figure 1D and Supplementary Table 2.6).

As stated in the methods section, the integrase peptide pool was not part of the vaccine construct but was used in the ELISpot assay for this trial to determine if the mechanism of vaccination could trigger non-specific changes in immune function. Data from week 0 to week 14 in participants receiving active drug showed no difference in the magnitude of the IFN-γ response to integrase, either for pooled group data or within the groups at these time points. In addition, at week 14 there were also no differences in magnitude comparing the groups (Figure 1E and Supplementary Figure 2.6).

With respect to favorable HLA genotypes (HLA B^*^27, B^*^57, B^*^58:01, B^*^81:01 and B^*^51) (18), 8/30 (27%) participants were found to have favorable HLA genotypes with 3 of these participants showing some change in the magnitude of the IFN-γ response from baseline. However, with so few responders overall a favorable HLA genotype is unlikely to have made a significant contribution in the context of this trial. There were 6 participants in the EP+IM group and 4 in the TC+IM group who did not receive active vaccine. Of these 3 participants showed an increase in the magnitude of the IFN-γ response at week 14. The implications of this are considered in the discussion section (Supplementary Figure 2.7). Our data did not show significant changes in CD4+ count in the group receiving active vaccination compared to placebo, as shown in the FIT-06 clinical trial (Supplementary Table 2.7).

### Viral Inhibition Assay: A High Proportion of Participants Were Shown to Inhibit Virus Pre-vaccination but Few Showed Variability at the Primary Endpoint

Only 5 participants had detectable endogenous virus (measure by p24) at baseline (week 0). 4 of 5 were shown to inhibit virus at week 0 with only 2 going on to show a change in log^10^ inhibition at week 14 (Supplementary Table 2.8). In the exogenous Viral inhibition assay 89% of participants inhibited virus at baseline, most commonly with U455 (clade A). At week 14, the majority (96%) of participants were shown to inhibit at least 1 virus (including in the placebo groups). However, there was no significant difference in log^10^ inhibition either between groups, or within groups comparing pre-vaccination (week 0) to primary endpoint (week 14) (Figures 1F,G). In addition, there was little change in the median breadth of viruses inhibited at week 14 in any group (Supplementary Figure 2.9).

### ICS: Significant Differences in Cytokine Production Were Observed in the TC+IM Group

**Figure 2:**
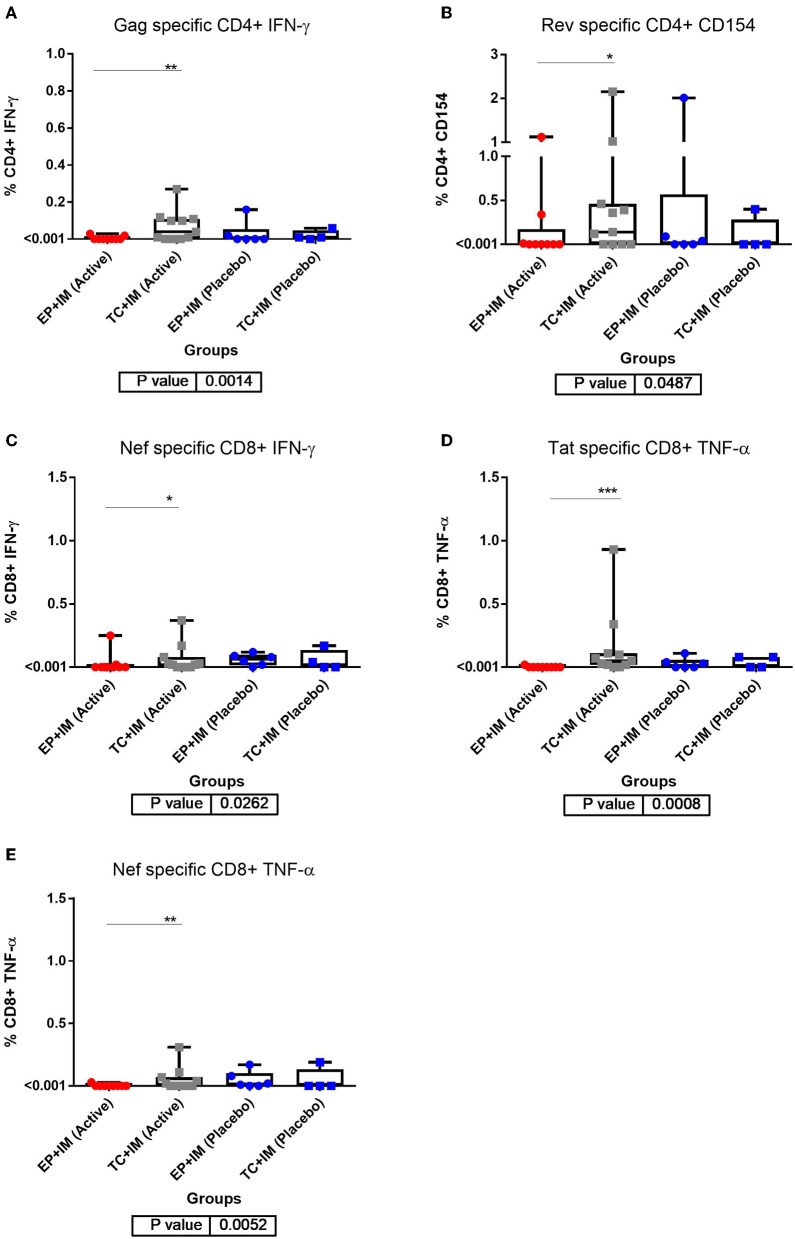
ICS analysis of T cell responses to DNA vaccination: **(A)** CD4+ IFN-ɤ response at primary endpoint (week 14) for all groups to Gag; **(B)** CD4+ CD154 response at primary endpoint (week 14) for all groups to Rev; **(C)** CD8+ IFN-ɤ response at primary endpoint (week 14) for all groups to Nef; **(D)** CD8+ TNF-α response at primary endpoint (week 14) for all groups to Tat; and **(E)** CD8+ TNF-α response at primary endpoint (week 14) for all groups to Nef. Statistical significance for all result is set at *p* = <0.05. ^*^p < 0.05, ^**^p <0.01, ^***^p<0.001.

The intracellular cytokine response detected in PBMC was detected by flow cytometry measuring antigen specific responses in both CD4+ and CD8+ T cells. Background levels of cytokine responses were found to be elevated in HIV positive volunteers compared to previous studies with HIV negative individuals. A significant increase in cytokine production was shown at the primary endpoint in the TC+IM group compared to the EP+IM group. This was observed for Tat and Nef specific CD8+ TNF-α response (*P* values of 0.0008 and 0.0052 respectively). IFN-γ production was shown to have significantly increased in CD4+ T cells after stimulation to Gag and in CD8+ T cells after Nef stimulation, with *P* values of 0.0014 and 0.0262 respectively (Figure 2). However, there was no statistical difference between TC+IM and the placebo control, this may reflect the smaller sample size. However, there was no clear trend overall which may be masked with the higher baseline levels of cytokines observed.

## Discussion

In this Phase I/II randomized controlled clinical trial we aimed to determine whether the mode of DNA vaccination could influence the quality and function of induced cellular immune responses compared to placebo, in an HIV positive cohort on ART. To the best of our knowledge this is the first human study of DNA vaccination using TC mode of vaccine delivery in people living with HIV. Overall, the vaccine was well-tolerated using both modes of delivery with no SAEs reported in the trial. In addition, despite reported pain associated with the EP procedure, participants considered this an acceptable mode of vaccine delivery as reported in previous clinical trials (11, 14).

In this cohort, participants were highly selected with nadir CD4+ counts ≥ 250 and undetectable viral loads. It is not entirely surprising to observe no change in response by IFN-γ. In addition, the variability of responses in the placebo group suggests that defining a “positive” response of >2-fold increase was too low. The presence of changes in response from baseline in placebo recipients could reflect the variability of background response to peptide pools in an HIV positive cohort, but could also be attributed to the actual procedures themselves (electroporation and transcutaneous skin surface stripping) creating inflammation and activating cells in the absence of active drug. The observation of similar changes in the placebo group suggests changes in the active groups are likely non-specific and not related to active vaccination.

With respect to the integrase peptide pool, with so few responders it is unlikely that vaccination has resulted in non-specific immune changes in either group at the primary endpoint. The presence of responders to this peptide pool in placebo recipients raises similar questions of background variation or inflammation created by the vaccination process.

To the best of our knowledge, this was the first trial using the transcutaneous route in combination with IM injection for DNA vaccination in an HIV positive cohort. In a previous trial using the same TC vaccine administration route, 6 HIV positive participants were vaccinated with Tetragrip (combined tetanus and influenza vaccine) and compared to the same vaccine delivered IM to 8 HIV positive patients. In the HIV positive volunteers receiving TC vaccination, there was a preferential amplification of vaccine-specific CD8+ cells (shown by ICS), with the authors concluding the results demonstrated this route of vaccination could affect the quality of the T cell response, albeit in small participant numbers (19).

In our previous CUTHIVAC 001 trial in HIV negative subjects, a comparison of week 0 and week 14 ICS responses within each group also showed a predominantly CD8+ response in the TC+IM group, specifically to IFN-γ, IL-2, and CD154 (11). However, these endpoint responses were of similar magnitude to pre-existing responses observed at baseline in our HIV cohort. These high baseline responses may have masked any effects of the vaccine in this current study.

As stated previously it is not possible to make direct comparisons on change in viral load in this trial with the FIT-06 clinical trial in South African infected clade C participants not on ART, as all participants in CUTHIVTHER were on suppressive ART. However, changes in CD4+ counts seen in FIT-06 were not replicated in this trial. One factor to consider here is this cohort had been established on effective ART, therefore may already have achieved effective restoration or near-restoration of immune function.

Only 5 participants had detectable endogenous virus production in the endogenous Viral inhibition assay. These data suggest for the majority of participants in this cohort of healthy HIV positive people on suppressive ART, the frequency of HIV positive cells that can be re-activated in culture is below the limit of detection. Nevertheless, 4/5 participants with endogenous virus displayed sufficient functioning CD8+ mediated immune responses to control *in vitro* viral replication even prior to vaccination.

In the exogenous Viral inhibition assay assessing the ability of CD8+ cells to inhibit exogenous virus, participants in each group were able to inhibit the panel of viruses at week 0 further supporting preservation of immune responses and specifically, CD8+ function. There was little change post vaccination in the ability to inhibit virus in any group, but participants shown to inhibit virus at week 14 also included placebo recipients. The modification of this assay needs further verification but could be a starting point for using the Viral inhibition assay in an HIV infected cohort.

Encouragingly these data show that HIV positive subjects with good nadir CD4+ counts and undetectable viral loads display potent CD8+ responses to HIV and functional viral inhibition in an *ex-vivo* assay. Neither IM DNA delivery with electroporation or transcutaneous immunization had a detectable impact on these high pre-vaccination responses, as measured by IFN-γ and ICS. Similarly, we were unable to detect an improvement in viral inhibition in the Viral inhibition assay suggesting that in this healthy HIV positive cohort on ART, CD8+ function remains relatively preserved. Nevertheless, the vaccine proved to be safe and tolerable. The inability to detect any beneficial response over and above the high levels of pre-existing immunity in this cohort does not mean the vaccine was inactive. Indeed, ICS analysis detected statistical differences between the TC+IM group and the EP+IM group but was underpowered to see any difference with the placebo matched control group. Future studies will be needed to determine whether DNA vaccination by either route can serve as an effective prime when combined with a boosting strategy including the use of viral vectors such as MVA (20). Additional benefit may be gained through strategies designed to refocus responses beneficial T-cell responses toward subdominant conserved regions of HIV-1 (21).

## Data Availability Statement

The datasets generated for this study are available on request to the corresponding author.

## Ethics Statement

The CUTHIVTHER 001 trial was conducted in compliance with UK Clinical Trial Regulations and within the principles of Good Clinical Practice (GCP). The study was approved by the National Research Ethics Service, York North East Research Ethics Committee (4/NE/1246, Eudract 2013-004023-37) and by the Medicines and Healthcare products Regulatory Authority (MHRA). All participants provided written informed consent after thorough counseling by the clinical trial team. The patients/participants provided their written informed consent to participate in this study.

## Author Contributions

RS, SM, AV, KR, DH, and BC oversaw and/or designed the study/immunogens. GH, MW, HR, and SM were involved in the day to day running and conduct of the study. GH, SD, AVC, SF, CY, AS, PH, BC, and RS were involved in laboratory analyses, interpretation of results, and drafting figures. GH, AC, and RS conducted the statistical analyses. GH and RS drafted the manuscript with editorial support and comment from AC, SD, PH, AV, SF, BC, and SM.

### Conflict of Interest

DH is employed by Ichor Medical Systems. KR is employed by FIT Biotech Ltd., Tampere, Finland. The remaining authors declare that the research was conducted in the absence of any commercial or financial relationships that could be construed as a potential conflict of interest.
