# Supplementary material for: The Safety and Immunogenicity of GTU®MultiHIV DNA Vaccine Delivered by Transcutaneous and Intramuscular Injection With or Without Electroporation in HIV-1 Positive Subjects on Suppressive ART
Source: Front Immunol. 2019 Dec 13;10:2911. doi: 10.3389/fimmu.2019.02911 (PMC6923267; doi:10.3389/fimmu.2019.02911)
Supplement: Supplementary file 1 [file Data_Sheet_1.docx]

Supplementary Material

Article Title

G. Haidari^1^, S. Day^1^, M. Wood^1^, H Ridgers^1^, A. Cope^1^, S. Fleck^2^, C. Yan^1^, K. Reijonen^3^, D. Hannaman^4^, A. Spentzou^1^, P. Hayes^5^, A. Vogt^6^, B. Combadiere^7^, A. Cook^8^, S. McCormack^8^, R J. Shattock^1^*

^1^Group of Mucosal Infection and Immunity, Imperial College London, Department of Medicine, London, United Kingdom

^2^London School of Hygiene and Tropical Medicine, London, United Kingdom

^3^FIT Biotech Ltd., Tampere, Finland

^4^Ichor Medical Systems Inc, San Diego, CA, United States

^5^Human Immunology Laboratory, International AIDS Vaccine Initiative, London, United Kingdom

^6^Clinical Research Center for Hair and Skin Science, Department of Dermatology and Allergy, Charité - Universitätsmedizin Berlin, Charitéplatz 1, 10117, Berlin, Germany

^7^Sorbonne Universités, UPMC Univ Paris 06, INSERM, U1135, CNRS, ERL 8255, Centre d'Immunologie et des Maladies Infectieuses (CIMI-Paris), 91 Boulevard de l'Hôpital, F-75013, Paris, France

^8^Medical Research Council Clinical Trials Unit at UCL, University College London, United Kingdom

*** Correspondence: Professor R Shattock r.shattock@imperial.ac.uk**

# Supplementary Data

**1.1 Transcutaneous Vaccination plus intramuscular vaccination (TC+IM)**

Participants in this group received a novel needle-free transcutaneous method to deliver 0.4mg (0.2mls) of the GTU^®^MultiHIV B DNA vaccine (or placebo) onto one upper arm below the deltoid muscle. To overcome the stratum corneum, the skin layer was disrupted using a process known as cyanoacrylate skin surface stripping (CSSS) where superglue was placed onto a predefined area on the upper arm (5 x 3.2cm), and adhesive tape place over the area. The glue was then left to dry for 20 minutes and stripped off. This technique opens the hair follicles and removes debris and sebum. The vaccine was then dripped on to the pre-defined site, massaged in using a finger cot for 1 minute, and allowed to absorb into the skin for 20 minutes. A clear bandage was placed over the arm and participants were advised not to wash for 24 hours and to avoid excessive sweating until the bandage was removed at the safety visit the following day. In addition, participants in this group also received a 2mg (1ml) IM injection of the vaccine (or placebo) into the left upper thigh to total at weeks 0, 4 and 12.

**1.2 Intramuscular Vaccination with Electroporation (EP+IM)**

In this group participants received a 2mg (1ml) IM injection the of GTU^®^MultiHIV B DNA (or placebo) into the left upper thigh at weeks 0, 4 and 12 followed by EP at the same site using the TriGrid integrated device. The disposable EP cartridge was loaded with GTU®MultiHIV B DNA vaccine (or placebo) then adjusted to one of three depth settings, corresponding to pre-defined ranges in skin fold thickness. The cartridge was then loaded into the handheld EP device and applied to the vastus lateralis muscle of the left thigh. IM administration was followed immediately by the application of electrical stimulation. The spacing of the TriGrid electrode array was 6 mm in a diamond-shaped configuration, and the electrical field was applied at an amplitude of 250 V/cm over a 40millisecond total duration, resulting in brief muscle contractions. All electroporation procedures were performed by the clinical research team.

**1.3 Optimisation of the integrase peptide pool**

An additional integrase peptide pool was added as a control to the assay as the presence of a peptide pool not included in the vaccine may give information on any non-specific changes occurring within T cells related to vaccination. In order to optimise this additional peptide pool, the integrase sequence was ordered in its entirety from NIH-AIDS Reagent Program, USA (see Supplementary Table 3.0). The relevant integrase peptides were then pooled together. The concentration of the integrase peptide pool used in the assay was 10µg/ml, with a final concentration in each well of 2.5µg/ml as per the other peptide pools.

The T cell ELISpot as described previously was conducted on 8 HIV uninfected donor PBMC in order to assess the background response to the integrase peptide pool. Apart from 1 donor which showed a high background response and is therefore excluded from analysis, all other donors had a negative response as expected (<55 SFU/M PBMC).

The additional integrase pool was used in the ELISpot assay at Week 0 (pre-vaccination), Week 14 (primary end point, 2 weeks after third and final visit), and Week 20 (final follow up visit).

**1.4 Viral Inhibition Assay**

*Can the CD8+ cells of vaccinated participants in the trial inhibit their endogenous virus?*

For this part of the assay, PBMCs from Week 0 and Week 14 for each participant were thawed as per previous with CD4+ targets and CD8+ effectors generated using the same steps involving bispecific antibodies CD3/8 and CD4/8 respectively (provided by Prof J Wong, Harvard Medical School, USA.) and IL-2.

At D7, both CD4+ and CD8+ cells were recovered. The CD4+ cells were not infected with exogenous virus. A 48 well plate was set up with 0.5million CD4 cells alone pre-vaccination (week 0), 0.5million CD4 cells post vaccination (week 14), and CD4+ and CD8+ cells pre and post vaccination. As described in previous assays, on days 3, 6 and 9 supernatant was removed and replaced with fresh R10/50 media. On day 13 supernatant was removed and frozen in duplicate ready for p24 ELISA analysis. The readings from this assay therefore represent the difference between endogenous virus pre-vaccination and at the primary end point as no exogenous virus was added.

For the p24 ELISA, all samples for Week 0 and Week 14 from participants were screened for the presence of endogenous virus, and only in those with virus at either time point was the ELISA conducted as per previous methods described.

*Can the CD8+ cells of vaccinated participants in the trial inhibit exogenous virus?*

For this part of the VIA the same PBMC vials used above from each participant at Week 0 and Week 14 were expanded and cultured for 7 days with the bispecific antibodies and IL-2. Maraviroc (MVC) at a concentration of 10µM was added into the culture and on days 3 and 6 when the culture volume was doubled, in order to block any endogenous virus.

At day 7, the cells were infected with exogenous virus. Due to cell number and availability, 3 viruses were used and these were all X4 viruses to prevent MVC (an R5 inhibitor) from blocking added virus. MVC continued to be added to the wells when supernatant was replaced with fresh medium (R10/50) in order to block any replication of endogenous virus. The p24 ELISAs were conducted using the same methods as previous.

**1.5** **Intracellular cytokine analysis**

T cell responses were evaluated by intracellular cytokine staining (ICS) with a panel for CD8+ and CD4+ antigen-specific responses.

ICS was performed on PBMC isolated on pre-vaccination (week 0), 2 weeks post-third vaccination (week 14) and 8 weeks post-third vaccination (week 20). In brief, cryopreserved PBMC were thawed and rested overnight in R10 media at 37°C, 5% CO_2_. After the overnight incubation, 1x10^6^ viable PBMC were stimulated for 6 hours at 37°C with 2.5 µg/mL of the overlapping peptide pool matching the vaccine (Gag, Nef, Rev, Tat and CTL, (Supplementary Table 3.0)) plus 1 µg/mL CD28/49d and CD107a BUV395 (H4A3) (BD Biosciences, San Diego, CA). Phorbol 12-myristate 13-acetate (PMA)/Ionomycin (Sigma-Aldrich, St. Louis, MO) and an additional integrase peptide pool were used as positive controls and R10 media with 0.25% DMSO was used as a negative control. Two hours into the stimulation, brefeldin A (eBioscience, San Diego, CA) and monensin (Biolegend, San Diego, CA) was added. Cells were stained with fixable viability dye eFluor780 (eBioscience, San Diego, CA), CD3 BV650 (Clone OKT3), CD4 PerCP-Cy5.5 (RPA-T4), CD8 AF700 (RPA-T8), IFN-γ AF488 (4S.B3), TNF-α PE-Cy7 (MAb11), IL-2 BV510 (MQ1-17H12), CD154 BV421 (24-31), Granzyme B AF647 (GB11) and Perforin PE (dG9) (BioLegend, San Diego, CA) to assess CD4/CD8 specific responses. Cells were stained with fixable viability dye eFluor780 at room temperature for 30 minutes, followed by washing and staining with 100 µL/well surface markers (CD3, CD4, CD8 in 2% human serum) for 20 minutes. Cells were washed and fixed with 100 µL/well IC fixation buffer (eBioscience) at room temperature for 20 minutes, then washed twice with IC Permeabilization buffer (eBioscience). Intracellular stain (IFN- γ, TNF-α, IL-2, CD154, granzyme B and perforin in assay buffer (PBS with 5% FCS and 0.05% sodium azide)) was added in a total of 100 µL/well at room temperature for 20 minutes, followed by 2 washes with assay buffer. The cells were resuspended in 3% paraformaldehyde and stored at 2-8°C for no longer than 18 hours prior to flow cytometry analysis on a Becton Dickinson Fortessa LSRII equipped with 20mW 355nm, 50mW 405nm, 50mW 488nm, 50mW 561nm, 20mW 633nm lasers and a ND1.0 filter in front of the FSC photodiode. Acquisition was set to record 50,000 live CD3+ lymphocytes after dead cell and doublet exclusion (FSC-A/W, SSC-A/W gating). Analysis was performed using FlowJo software (Treestar, Ashland, OR). Data was expressed as percentage of total CD4+ or CD8+ cells. Boolean gating was used to determine polyfunctionality.

# Supplementary Figures and Tables

Supplementary Table 2.1: Inclusion/Exclusion criteria for CUTHIVTHER 001

| **INCLUSION CRITERIA** |
| --- |
| Men and women aged between 18-45 |
| BMI between 19-30 |
| Willing and able to give informed written consent |
| HIV Clade B infection documented by confirmed Ab test |
| Confirmed on 2 separate occasions in the 6 months prior to enrolment to have viral load <50 copies HIV RNA/ml whilst on ART |
| Nadir CD4+ ≥250 and screening CD4+ ≥200 lymphocytes |
| If heterosexually active female using an effective method of contraception (includes consistent condom use). If heterosexually active male using effective method with partner |

| **EXCLUSION CRITERIA** |
| --- |
| Pregnant or lactating |
| Use of topical treatment on injection or application site within last 4 weeks |
| No UV tanning sessions or strong sun exposure within 4 weeks prior to study |
| Clinically relevant abnormality on history or examination including:   - History of seizure disorder - Severe eczema - Liver disease including active Hep B (HepBsAg pos) or C (PCR positive) - Any skin condition which may interfere with injection site - Haematological, metabolic, gastrointestinal, cardiac disorders including abnormal ECG |
|  |
| Excessive terminal hair growth on investigational areas |
| Grade 2 or above abnormal laboratory parameters (including conjugated hyperbilirubinaemia) |
| Previous severe reaction to vaccination or receipt of live attenuation vaccine in last 60 days |

Supplementary Figure 2.2: a) Vaccine schedule and b) Consort diagram outlining participant flow in the 2 groups

a)

b)

Participants screened (N=36)

(N=

Enrolled

(N=30)

(N=

**Excluded (N=6)**

Withdrew consent (N=2)

Abnormal laboratory screening results (N=2)

Other exclusion criteria (N=2)

Randomisation

**Group 1 IM+EP (N=15)**

Active N=9 Placebo N=6

Week 0

**Group 1 IM+EP**

Week 4

**Group 2 IM+TC**

Week 4

**Group 1 IM+EP**

Week 12

**Group 2 IM+TC**

Week 12

Discontinued intervention, received 2 vaccinations, but remained in follow-up at Week 12 (N=1)

**Group 2 TC+EP (N=15)**

Active N=11 Placebo N=4

Week 0

Supplementary Table 2.3 HIV related characteristics (all participants enrolled)

| **HIV related characteristics** | **All (n=30)** |
| --- | --- |
| **Sexuality**  **MSM**  **Bisexual** | 29 (97%)  1 (3%) |
| **Regular partner** | 17 (57%) |
| **Years since HIV diagnosis**  **Median**  **IQR**  **Range** | 3  1.6-7  0.9-11.5 |
| **CD4 nadir (cells/mm^3^)**  **Median**  **IQR**  **Range** | 346  300-516  260-968 |
| **Last VL <50 copies/ml** | 30 (100%) |
| **Time on current ART regimen (years)**  **Mean**  **IQR**  **Range** | 2.18  1.14-2.90  0.6-7.18 |
| **ART regimen at enrolment**  **NRTI + NNRTI**  **NRTI + PI**  **NRTI + INI**  **Other** | 20 (67%)  5 (17%)  4 (13%)  1 (3%) |

Supplementary Table 2.4: Summary of unsolicited adverse events by group with relationship to vaccine shown. All unsolicited adverse events were documented as grade 1 or 2.

| **Safety:**  **Unsolicited Adverse events** | **EP+IM**  **(active)**  **N=9** | **EP+IM**  **(placebo)**  **N=6** | **TC+IM**  **(active)**  **N=11** | **TC+IM**  **(placebo)**  **N=4** | **Total**  **(%)** |
| --- | --- | --- | --- | --- | --- |
| Definitely related to vaccine | 0 | 0 | 0 | 0 | 0 |
| Probably related | 0 | 0 | 14 | 2 | 16 (19%) |
| Possibly related | 4 | 8 | 19 | 4 | 35 (43%) |
| Unlikely related to vaccine | 10 | 9 | 6 | 2 | 27 (33%) |
| Not related | 1 | 0 | 3 | 0 | 4 (5%) |
| **Total** | **15** | **17** | **42** | **8** | **82 (100%)** |

Supplementary Table 2.5: Tolerability data for EP+IM active and placebo groups showing mean and median pain scores (0 = no pain, 10 = worst pain ever) with interquartile range immediately after EP and 30 minutes later for each vaccination.

|  | **EP+IM (active)**  **(n=9)** | **EP+IM (placebo) (n=6)** | **EP+IM (combined) (n=15)** |
| --- | --- | --- | --- |
| **Pain at vaccination 1** |  |  |  |
| **Immediately after EP**  **Mean**  **Median (IQR)  30 mins after EP**  **Mean**  **Median (IQR)** | 5 4 (4-6)  1.2  1 (1-2) | 5.8 6 (5-6)  2.5  2.5 (2-3) | 5.3  5 (4-6)  1.7  2 (1-3) |
| **Pain at vaccination 2** |  |  |  |
| **Immediately after EP**  **Mean**  **Median (IQR)  30 mins after EP**  **Mean**  **Median (IQR)** | 4.5 5 (4-6)  1.9  1 (1-3) | 5.7 6 (5-6)  1.5  1.5 (0-2) | 5  6 (4-6)  1.7  1 (0-3) |
| **Pain at vaccination 3** |  |  |  |
| **Immediately after EP**  **Mean**  **Median (IQR)  30 mins after EP**  **Mean**  **Median (IQR)** | 4.4 3.5 (3-6.5)  0.5  0 (0-1) | 5 6 (5-6)  0.7  0.5 (0-1) | 4.6  4 (3-6)  0.6  0 (0-1) |

Supplementary Table 2.6 Summary of IFN-ɣ responses at week 0 (pre-vaccination) by peptide and group.

| **Week 0**  **Responders** | **EP+IM**  **(Active)** | **EP+IM**  **(Placebo)** | **TC+IM**  **(Active)** | **TC+IM**  **(Placebo)** | **TOTAL** |
| --- | --- | --- | --- | --- | --- |
| ***Rev*** | 2 |  | 1 |  | **3/30 (10%)** |
| ***Tat*** | 2 |  |  |  | **2/30 (7%)** |
| ***Nef*** | 8 | 4 | 8 | **4** | **24/30 (80%)** |
| ***Gag*** | 7 | 6 | 9 | **4** | **26/30 (87%)** |
| ***CTL*** | 5 | 3 | 4 | **0** | **12/30 (40%)** |

Supplementary Figure 2.6. Magnitude of the IFN-ɣ response to the integrase peptide pool for EP+IM and TC+IM groups showing no statistically significant response at pre and post vaccination time points.

Supplementary Figure 2.7: Positive responders by IFN-ɣ in placebo recipients where each dot joined by a line is the participant’s response to that particular peptide pool from week 0 to week 14. Positive responses are indicated by colored dots and labelled according to the responding peptide pool.

Supplementary Table 2.7: Mean CD4+ counts with standard deviations shown by group at week 0 and week 14

| **Group** | **Mean CD4+ count Week 0** | **Standard Deviation Week 0** | **Mean CD4+ count Week 14** | **Standard Deviation Week 14** |
| --- | --- | --- | --- | --- |
| EP+IM Active | 807 | 220 | 844 | 265 |
| EP+IM Placebo | 818 | 140 | 767 | 289 |
| TC+IM Active | 709 | 224 | 710 | 200 |
| TC+IM Placebo | 677 | 108 | 659 | 137 |
| **Total (all grps)** | 753 |  | 745 |  |

Supplementary Table 2.8. Endogenous virus production measured by p24 concentration in 5/30 participants at any time point with log^10^ inhibition shown at week 0 and week 14. A change was seen in 2 participants: CT016 (EP+IM active group) and CT002 (TC+IM placebo group).

| **Participant** | **Group** | **Week 0**  **Log^10^ inhibition** | **Week 14**  **Log^10^ inhibition** |
| --- | --- | --- | --- |
| CT016 | EP+IM  (active) | 1.93 | 3.26 |
| CT007 | TC+IM  (active) | 2.31 | 2.31 |
| CT009 | EP+IM  (placebo) | 2.15 | 2.26 |
| CT002 | TC+IM  (placebo) | 0.97 | 2.68 |
| CT012 | TC+IM  (placebo) | 3.90 | 3.90 |

Supplementary Table 2.9. Peptide pools used for T cell ELISpot and ICS assays

| **Rev** | **Tat** | **Nef** | **P17/p24** | **P17/p24 (cont)** |
| --- | --- | --- | --- | --- |
| AGRSGDSDEELLKTV  GDSDEELLKTVRLIK  EELLKTVRLIKFLYQ  KTVRLIKFLYQSNPP  LIKFLYQSNPPPSNE  LYQSNPPPSNEGTRQ  SNPPPSNEGTRQARRN  SNEGTRQARRNRRRR  TRQARRNRRRRWRER  RRNRRRRWRERQRQI  RRRWRERQRQIRSIS  RERQRQIRSISERIL  RQIRSISERILSTFL  SISERILSTFLGRPA  RILSTFLGRPAEPVP  TYLGRPAEPVPLQLP  RPAEPVPLQLPPLER  PVPLQLPPLERLTLD  LQLPPLERLTLDCSED  LERLTLDCSEDCGNS  TLDCSEDCGNSGTQG  CSEDCGNSGTQGVGSP  GNSGTQGVGSPQVLV  TQGVGSPQVLVESPA  GSPQVLVESPAVLEP  VLVESPAVLEPGTKE | EPVDPRLEPWKHPGS  PRLEPWKHPGSQPRT  PWKHPGSQPRTPCTN  PGSQPRTPCTNCYCK  PRTPCTNCYCKKCCL  CTNCYCKKCCLHCQV  YCKKCCLHCQVCFTR  CCLHCQVCFTRKGLG  CQVCFTRKGLGISYG  FTRKGLGISYGRKKR  GLGISYGRKKRRQRR  SYGRKKRRQRRRAPQ  KKRRQRRRAPQDSQT  RQRRRAPQDSQTHQVS  APQDSQTHQVSLPKQ  SQTHQVSLPKQPSSQ  HQVSLPKQPSSQQRGD  PKQPSSQQRGDPTGP  SSQQRGDPTGPKKSK  RGDPTGPKKSKKKVE  TGPKKSKKKVERETE  KSKKKVERETEADPF  KVERETEADPFDAAV | TSVGKWSKCSGWPTV  KWSKCSGWPTVRERM  CSGWPTVRERMKQAE  PTVRERMKQAEPEPA  ERMKQAEPEPAADGV  QAEPEPAADGVGAAS  EPAADGVGAASRDLE  DGVGAASRDLEKHGA  AASRDLEKHGAITSS  DLEKHGAITSSNTAT  HGAITSSNTATNNAA  TSSNTATNNAACAWL  TATNNAACAWLEAQE  CAWLEAQEEEEVGFP  AWLEAQEEEEVGFPV  AQEEEEVGFPVRPQV  EEVGFPVRPQVPLRP  FPVRPQVPLRPMTYK  PQVPLRPMTYKGALD  LRPMTYKGALDLSHF  TYKGALDLSHFLKEK  ALDLSHFLKEKGGLE  SHFLKEKGGLEGLIY  KEKGGLEGLIYSPKR  GLEGLIYSPKRQEIL  LIYSPKRQEILDLWV  PKRQEILDLWVYHTQ  RQDILDLWVYHTQGYF  LWVYHTQGYFPDWQN  HTQGYFPDWQNYTPG  YFPDWQNYTPGPGVR  WQNYTPGPGVRYPLT  TPGPGVRYPLTFGWC  GVRYPLTFGWCFKLV  PLTFGWCFKLVPVEP  GWCFKLVPVEPDEEE  KLVPVEPDEEENSSL  VEPDEEENSSLLHPA  EEENSSLLHPASLHG  SSLLHPASLHGTEDT  HPASLHGTEDTEREV  LHGTEDTEREVLKWK  EDTEREVLKWKFDSH  EVLVWKFDSRLAFHH  KWKFDSHLAFHHKAR  DSHLAFHHKARELHP  AFHHKARELHPEYYK  KARELHPEYYKDCKL | GARASVLSGGELDKW  SVLSGGELDKWEKIR  GGELDKWEKIRLRPG  DKWEKIRLRPGGKKK  KIRLRPGGKKKYQLK  RPGGKKKYQLKHIVW  KKKYQLKHIVWASRE  QLKHIVWASRELERF  IVWASRELERFAVNP  SRELERFAVNPGLLE  ERFAVNPGLLETSEG  VNPGLLETSEGCRQI  LLETSEGCRQIMGQL  SEGCRQIMGQLQPSL  RQIMGQLQPSLQTGS  GQLQPSLQTGSEELR  PSLQTGSEELRSLYN  TGSEELRSLYNTVAT  ELRSLYNTVATLYCV  LYNTVATLYCVHQKI  VATLYCVHQKIEVKD  YCVHQKIEVKDTKEA  HQKIEVKDTKEALDKV  VKDTKEALDKVEEEQ  KEALDKVEEEQNNSK  DKVEEEQNNSKKKAQ  EEQNNSKKKAQQEAA  NSKKKAQQEAADAGN  KAQQEAADAGNRNQV  EAADAGNRNQVSQNY  AGNRNQVSQNYPIVQ  SQVSQNYPIVQNLQG  SQNYPIVQNLQGQMVH  IVQNLQGQMVHQAIS  LQGQMVHQAISPRTL  MVHQAISPRTLNAWV  AISPRTLNAWVKVVE  RTLNAWVKVVEEKAF  AWVKVVEEKAFSPEV  VVEEKAFSPEVIPMF  KAFSPEVIPMFSALS  PEVIPMFSALSEGAT  PMFSALSEGATPQDL  ALSEGATPQDLNTML  GATPQDLNTMLNTVG  PQDLNTMLNTVGGHQA  TMLNTVGGHQAAMQM  TVGGHQAAMQMLKET  HQAAMQMLKETINEE  MQMLKETINEEAAEW  KETINEEAAEWDRLH  INEEAAEWDRLHPVHA  AEWDRLHPVHAGPIA | RLHPVHAGPIAPGQM  VHAGPIAPGQMREPR  PIAPGQMREPRGSDI  GQMREPRGSDIAGTT  EPRGSDIAGTTSTLQ  SDIAGTTSTLQEQIG  GTTSTLQEQIGWMTN  TLQEQIGWMTNNPPI EQIGWMTNNPPIPVGE MTNNPPIPVGEIYKR  PPIPVGEIYKRWIIL  VGEIYKRWIILGLNK  YKRWIILGLNKIVRM  IILGLNKIVRMYSPT  LNKIVRMYSPTSILD  VRMYSPTSILDIKQG  SPTSILDIKQGPKEP  ILDIKQGPKEPFRDY  KQGPKEPFRDYVDRF  KEPFRDYVDRFYKTL  RDYVDRFYKTLRAEQ  DRFYKTLRAEQATQE  KTLRAEQATQEVKNW  AEQATQEVKNWMTET  TQEVKNWMTETLLVQ  KNWMTETLLVQNANP  TETLLVQNANPDCKT  LVQNANPDCKTILKA  ANPDCKTILKALGPA  CKTILKALGPAATLE  LKALGPAATLEEMMT  GPAATLEEMMTACQG  TLEEMMTACQGVGGP  MMTACQGVGGPGHKA  CQGVGGPGHKARVLP |
| **CTL** |  |  |  |  |
| ITLWQRPLVTIKIGG  ALIEICTEMEKEGKIS  CTEMEKEGKISKIGP  AGLKKKKSVTVLDVG  KKKSVTVLDVGDAYF  VTVLDVGDAYFSVPL  DVGDAYFSVPLDKDF  AYFSVPLDKDFRKYT  VPLDKDFRKYTAFTI  KDFRKYTAFTIPSIN  WKGSPAIFQSSMTKI  KQNPDIVIYQYMDDL  DIVIYQYMDDLYVGS  TVQPIVLPEKDSWTV  LVGKLNWASQIYAGI  LNWASQIYAGIKVKQ  REILKEPVHGVYYDP  EKEPIVGAETFYVDG  IVGAETFYVDGAANR  AGNLWVTVYYGVPV  LWVTVYYGVPVWKEA  VYYGVPVWKEATTTL  VERYLRDQQLLGIWGCA  INQMLRGPGRAFVTI |  |  |  |  |

Supplementary Figure 2.9: Breadth of viruses inhibited shown on graph as weeks 0 and 14 with median and interquartile range.
